# Supplementary material for: Aberrant calcium channel splicing drives defects in cortical differentiation in Timothy syndrome
Source: eLife. 2019 Dec 23;8:e51037. doi: 10.7554/eLife.51037 (PMC6964969; doi:10.7554/eLife.51037)
Supplement: Supplementary file 1. [file elife-51037-supp1.docx]

| **Key Resources Table** | | | | |
| --- | --- | --- | --- | --- |
| **Reagent type (species) or resource** | **Designation** | **Source or reference** | **Identifiers** | **Additional information** |
| mouse strain (*M. musculus*) | timed pregnant C57/bl6 dams, postnatal pups and adult mice | Charles River Laboratories | C57BL/6NCrl, Strain Code #027; IMSR Cat# CRL:027; RRID:IMSR_CRL:027 | for calcium imaging of wild-type NPCs, and Ca_v_1.2 protein expression and mRNA splicing analyses |
| mouse strain (*M. musculus*) | Timed pregnant Swiss Webster dams | Charles River Laboratories | Crl:CFW(SW), Strain Code #024; IMSR Cat# CRL:024; RRID:IMSR_CRL:024 | for *in utero* gain of function experiments |
| mouse strain (*M. musculus*) | Ca_v_1.2 mice: *Cacna1c*^flx/+^ and *Cacna1c*^+/-^ mice | Gomez-Ospina N, et al. 2013 (PMID: 23613729) | Floxed mice: *Cacna1c*^tm3Hfm^/J (Stock # 024714 \| Ca_v_1.2 loxP); IMSR Cat# JAX:024714; RRID:IMSR_JAX:024714 | for *in utero* loss of function experiments; *Cacna1c*^flx/+^ mice were bred to *Cacna1c*^+/-^ mice and electroporation of pCAG-cre:eGFP was carried out on the embryos from the cross; the *Cacna1c* null allele was created by ubiquitously deleting the floxed Ca_v_1.2 exons using CMV-CRE transgenic mice |
| cell line (*M. musculus*) | Neuro2A neuroblastoma cells | ATCC | ATCC Cat# CCL-131; RRID:CVCL_0470 | calcium imaging of *in utero* expression constructs |
| cell line (*Homo sapiens*) | HEK293 | ATCC | ATCC Cat# CRL-1573; RRID:CVCL_0045 | used to confirm co-expression of GFP and Ca_v_1.2 from *in utero* expression constructs |
| cell lines (*Homo sapiens*) | control and TS iPSC lines | Pasca SP, et al. 2011 (PMID: 22120178) | control (IM23-9, NH1-1, NH2-6), TS (T7643-5, T7643-7, T7643-32, T9862-42) | all lines used were generated and previously validated, tested for mycoplasma contamination and maintained mycoplasma free |
| biological samples (*Homo sapiens*) | RNA, frontal lobe | Biochain | #R1244051 (multiple specimens at different ages), #R1234051; Biochain, RRID:SCR_013548 | RNA from frontal cortex specimens at different developmental timepoints |
| antibody | anti-Calcium Channel, Voltage Gated α1C (rabbit polyclonal) | Millipore | Millipore Cat# AB5156; RRID:AB_91704 | IF (1:150), WB (1:500) |
| antibody | anti-PAX6  (mouse monoclonal) | Developmental Studies Hybridoma Bank (DSHB) | DSHB Cat# pax6, RRID:AB_528427 | IF (1:100) |
| antibody | anti-GFP (chicken polyclonal) | Abcam | Abcam Cat# ab13970; RRID:AB_300798 | IF (1:500) |
| antibody | anti-CTIP2 (rat monoclonal) | Abcam | Abcam Cat# ab18465; RRID:AB_2064130 | IF (1:500) |
| antibody | anti-SATB2 (mouse monoclonal), clone SATBA4B10 | Abcam | Abcam Cat# ab51502; RRID:AB_882455 | IF (1:100) |
| antibody | anti-PAX6 (rabbit polyclonal) | Biolegend | Cat #PRB-278P (formerly Covance #PRB-278P; Covance RRID:AB_291612) | IF (1:200) |
| antibody | anti-TBR2/Eomes [Dan11mag] (rat monoclonal) | Thermo Fisher | Thermo Fisher Scientific Cat# 14-4875-82; RRID:AB_11042577 | IF (1:500) |
| antibody | anti-6x His tag [4D11] (mouse monoclonal) | Abcam | Abcam Cat# ab5000; RRID:AB_304722 | IF (1:100) |
| antibody | anti-TBR1 (rabbit polyclonal) | Abcam | Abcam Cat# ab31940; RRID:AB_2200219 | IF (1:200) |
| antibody | anti-GAPDH (mouse monoclonal) | Sigma | Sigma-Aldrich Cat# G8795; RRID:AB_1078991 | WB (1:1000) |
| other | nuclear counterstain Hoechst 33258 | Molecular probes | Thermo Fisher Scientific Cat# H3569; RRID:AB_2651133 | IF (1:10,000) |
| recombinant DNA reagent | DHP-Ca_v_1.2 in pcDNA4/HisMax vector | Dolmetsch RE, et al. 2001 (PMID: 11598293) |  | Ca_v_1.2 expression construct |
| recombinant DNA reagent | DHP-TS-Ca_v_1.2 in pCDNA4/His Max vector | Krey JF, et al. 2013 (PMID: 23313911) |  | Ca_v_1.2 expression construct |
| recombinant DNA reagent | DHP-TS-4EQ-Ca_v_1.2 in pCDNA4/His Max vector | Krey JF, et al. 2013 (PMID: 23313911) |  | Ca_v_1.2 expression construct |
| recombinant DNA reagent | pDEST-β1b | Park CY, et al. 2010 (PMID: 20929812) |  | Ca_v_1.2 auxiliary subunit β1b expression construct |
| recombinant DNA reagent | pDEST-α2δ1 | Park CY, et al. 2010 (PMID: 20929812) |  | Ca_v_1.2 auxiliary subunit α2δ1 expression construct |
| recombinant DNA reagent | pCAGIG (plasmid) | kindly provided by Dr. Connie Cepko through Addgene | Addgene plasmid 11159; RRID:Addgene_11159 | backbone for expression of Ca_v_1.2 cDNAs |
| recombinant DNA reagent | pCAGIG- Cav1.2-IRES-EGFP | This paper |  | *in utero* expression construct |
| recombinant DNA reagent | pCAGIG-TS-Cav1.2-IRES-EGFP | This paper |  | *in utero* expression construct |
| recombinant DNA reagent | pCAGIG-WT-4EQ-Cav1.2-IRES-EGFP | This paper |  | *in utero* expression construct |
| recombinant DNA reagent | pCAGIG-TS-4EQ-Cav1.2-IRES-EGFP | This paper |  | *in utero* expression construct |
| recombinant DNA reagents | pCAG-cre:EGFP (plasmid) | kindly provided by Dr. Connie Cepko through Addgene | Addgene plasmid 13776; RRID:Addgene_13776 | expression plasmid, cre:EGFP |
| strain, strain background (*E. coli*) | XL10-Gold Ultracompetent Cells | Agilent | 200315 | ultracompetent cells |
| sequence based reagent | mouse *Cacna1c-exon 8*-fwd | This paper; Portmann T, et al. 2014 (PMID: 24794428) | PCR primers | 5'-CTGACGGTGTTCCAGTGTATCA-3' |
| sequence based reagent | mouse *Cacna1c-exon 8*-rev | This paper; Portmann T, et al. 2014 (PMID: 24794428) | PCR primers | 5'-ACTCATAGCCCATAGCGTCTTG-3' |
| sequence based reagent | mouse *Cacna1c-exon 8A*-fwd | This paper; Portmann T, et al. 2014 (PMID: 24794428) | PCR primers | 5'-GTCAATGATGCCGTAGGAAG-3' |
| sequence based reagent | mouse *Cacna1c-exon 8A*-rev | This paper; Portmann T, et al. 2014 (PMID: 24794428) | PCR primers | 5'-CCGCTAAGAACACCGAGAA-3' |
| sequence based reagent | mouse *Pax6*-fwd | PrimerBank | PCR primers, PrimerBank ID #1405745a1 | 5'-TACCAGTGTCTACCAGCCAAT-3' |
| sequence based reagent | mouse-*Pax6*-rev | PrimerBank | PCR primers, PrimerBank ID #1405745a1 | 5'-TGCACGAGTATGAGGAGGTCT-3' |
| sequence based reagent | mouse-*Nestin*-fwd | PrimerBank | PCR primers, PrimerBank ID #15011851a1 | 5’-CCCTGAAGTCGAGGAGCTG-3’ |
| sequence based reagent | mouse-*Nestin*-rev | PrimerBank | PCR primers, PrimerBank ID #15011851a1 | 5’-CTGCTGCACCTCTAAGCGA-3’ |
| sequence based reagent | mouse-*Gad67*-fwd | PrimerBank | PCR primers, PrimerBank ID #31982847a1 | 5'-CACAGGTCACCCTCGATTTTT-3' |
| sequence based reagent | mouse-*Gad67*-rev | PrimerBank | PCR primers, PrimerBank ID #31982847a1 | 5'-ACCATCCAACGATCTCTCTCATC-3' |
| sequence based reagent | Mouse-*Gapdh*-fwd | PrimerBank | PCR primers, PrimerBank ID #6679937a1 | 5’-AGGTCGGTGTGAACGGATTTG-3’ |
| sequence based reagent | Mouse-*Gapdh*-fwd | PrimerBank | PCR primers, PrimerBank ID #6679937a1 | 5’-TGTAGACCATGTAGTTGAGGTCA-3’ |
| sequence based reagent | human *CACNA1C-exon 8*-fwd | This paper | PCR primers | 5'-ACGCTATGGGCTATGAGTTACC-3' |
| sequence based reagent | human *CACNA1C-exon 8*-rev | This paper | PCR primers | 5'-GGCCTTCTCCCTCTCTTTG-3' |
| sequence based reagent | human *CACNA1C-exon 8A*-fwd | This paper | PCR primers | 5'-TTTGACAACTTTGCCTTCGC-3' |
| sequence based reagent | human *CACNA1C-exon 8A*-rev | This paper | PCR primers | 5'-TCCCTTCCTACGGCATCATT-3' |
| sequence based reagent | human *NCAM*-fwd | PrimerBank | PCR primers, PrimerBankID #10834990a1 | 5’-ACATCACCTGCTACTTCCTGA-3’ |
| sequence based reagent | human *NCAM*-rev | PrimerBank | PCR primers, PrimerBankID #10834990a1 | 5’-CTTGGACTCATCTTTCGAGAAGG-3’ |
| sequence based reagent | human *GAPDH*-fwd | PrimerBank | PCR primers, PrimerBankID #7669492a3 | 5’-CATGAGAAGTATGACAACAGCCT-3 |
| sequence based reagent | human *GAPDH*-rev | PrimerBank | PCR primers, PrimerBankID #7669492a3 | 5’-AGTCCTTCCACGATACCAAAGT-3’ |
| sequence based reagent | mouse *Cacna1c-exon 8* LNA *ISH* probe | Exiqon | LNA *ISH* probe | 5'-biotin-A+CT CA+T AGC +CCA TAG CGT +CT-3'  (+ signs indicate locations of LNA bases) |
| sequence based reagent | mouse *Cacna1c-exon 8A* LNA *ISH* probe | Exiqon | LNA *ISH* probe | exon 8A: 5'-dig-AGT+CC+CTTCGTA+CGGCATCA-3'  (+ signs indicate locations of LNA bases) |
| sequence based reagent | sense LNA *ISH* probe | Exiqon | LNA *ISH* probe | 5'-biotin-TGCGA+TA+CC+CGATA+CT+CAAC-3'  (+ signs indicate locations of LNA bases) |
