# Supplementary material for: Aberrant calcium channel splicing drives defects in cortical differentiation in Timothy syndrome
Source: eLife. 2019 Dec 23;8:e51037. doi: 10.7554/eLife.51037 (PMC6964969; doi:10.7554/eLife.51037)
Supplement: Transparent reporting form [file elife-51037-transrepform.docx]

***eLife’s* transparent reporting form**

We encourage authors to provide detailed information *within their submission* to facilitate the interpretation and replication of experiments. Authors can upload supporting documentation to indicate the use of appropriate reporting guidelines for health-related research (see [EQUATOR Network](http://www.equator-network.org/%20)), life science research (see the [BioSharing Information Resource](https://biosharing.org/)), or the [ARRIVE guidelines](http://www.plosbiology.org/article/info:doi/10.1371/journal.pbio.1000412) for reporting work involving animal research. Where applicable, authors should refer to any relevant reporting standards documents in this form.

If you have any questions, please consult our Journal Policies and/or contact us: [editorial@elifesciences.org](mailto:editorial@elifesciences.org).

**Sample-size estimation**

- You should state whether an appropriate sample size was computed when the study was being designed
- You should state the statistical method of sample size computation and any required assumptions
- If no explicit power analysis was used, you should describe how you decided what sample (replicate) size (number) to use

Please outline where this information can be found within the submission (e.g., sections or figure legends), or explain why this information doesn’t apply to your submission:

Sample sizes were empirically determined based on data from similar previous studies in our field, in order to provide sufficient power for statistical comparisons. This statement can be found in the Methods section under ‘Statistics’, and the sample size for each experiment is stated in the corresponding figure legends.

**Replicates**

- You should report how often each experiment was performed
- You should include a definition of biological versus technical replication
- The data obtained should be provided and sufficient information should be provided to indicate the number of independent biological and/or technical replicates
- If you encountered any outliers, you should describe how these were handled
- Criteria for exclusion/inclusion of data should be clearly stated
- High-throughput sequence data should be uploaded before submission, with a private link for reviewers provided (these are available from both GEO and ArrayExpress)

Please outline where this information can be found within the submission (e.g., sections or figure legends), or explain why this information doesn’t apply to your submission:

The number of replicates for each experiment are included in the corresponding figure legends, as well as in the Methods section. Briefly, for splicing analysis in the mouse cortex, RNA from 3 individual mouse cortices from 2 independent litters was used. For splicing analysis in human iPSC-derived neural cells, we used the following: control NPCs, 2 subjects and 1 H9 hES line (4 lines total); control neurons, 2 subjects and 1 H9 hES line (5 lines total); and TS NPCs and neurons, 2 subjects (four lines total). Results are reported as mean +/- sem across lines after lines were averaged across independent differentiations. For WB expression analysis of Cav1.2 in mouse cortex, protein was extracted from four pooled embryonic mouse cortices at E14 and at E18 from two separate litters. For *in utero* electroporation experiments, quantifications were performed on three to six sections per animal spanning caudal to rostral lateral cortex two to four days after electroporation, from at least three animals per experimental group with >100 EGFP+ electroporated cells in anatomically-matched regions of interest. At least two separate litters per experimental group were assessed.

**Statistical reporting**

- Statistical analysis methods should be described and justified
- Raw data should be presented in figures whenever informative to do so (typically when N per group is less than 10)
- For each experiment, you should identify the statistical tests used, exact values of N, definitions of center, methods of multiple test correction, and dispersion and precision measures (e.g., mean, median, SD, SEM, confidence intervals; and, for the major substantive results, a measure of effect size (e.g., Pearson's r, Cohen's d)
- Report exact p-values wherever possible alongside the summary statistics and 95% confidence intervals. These should be reported for all key questions and not only when the p-value is less than 0.05.

Please outline where this information can be found within the submission (e.g., sections or figure legends), or explain why this information doesn’t apply to your submission:

Details regarding statistical analysis is included under the ‘Statistics’ subheading in the Methods section, as well as in each corresponding figure legend. Individual data points are included for all plots.

(For large datasets, or papers with a very large number of statistical tests, you may upload a single table file with tests, Ns, etc., with reference to sections in the manuscript.)

**Group allocation**

- Indicate how samples were allocated into experimental groups (in the case of clinical studies, please specify allocation to treatment method); if randomization was used, please also state if restricted randomization was applied
- Indicate if masking was used during group allocation, data collection and/or data analysis

Please outline where this information can be found within the submission (e.g., sections or figure legends), or explain why this information doesn’t apply to your submission:

Details regarding group allocation for each experiment can be found in the corresponding methods section.

**Additional data files (“source data”)**

- We encourage you to upload relevant additional data files, such as numerical data that are represented as a graph in a figure, or as a summary table
- Where provided, these should be in the most useful format, and they can be uploaded as “Source data” files linked to a main figure or table
- Include model definition files including the full list of parameters used
- Include code used for data analysis (e.g., R, MatLab)
- Avoid stating that data files are “available upon request”

Please indicate the figures or tables for which source data files have been provided:

Source data have been provided for Figures 1-4 (Fig 1a,c,d,e,f,h; Fig 2i,n; Fig 3d,h; Fig 4d,f), as well as Figure 1—figure supplement 1a,d,e,f,i,j.
